# Preconditioning Effect of High-Intensity Interval Training (HIIT) and Berberine Supplementation on the Gene Expression of Angiogenesis Regulators and Caspase-3 Protein in the Rats with Myocardial Ischemia-Reperfusion (IR) Injury

**DOI:** 10.1155/2020/4104965

**Published:** 2020-09-04

**Authors:** Parisa Banaei, Farzad Nazem, Afshin Nazari, Arash Arjomand

**Affiliations:** ^1^Department of Sport Physiology, Faculty of Physical Education and Sport Sciences, Bu-Ali Sina University, Hamedan 6517838695, Iran; ^2^Department of Sport Physiology, Faculty of Physical Education and Sport Sciences, Bu-Ali Sina University, Hamedan 6517838695, Iran; ^3^Department of Physiology, Razi Herbal Medicine Research Center, Lorestan University of Medical Sciences, Khorramabad 6813846464, Iran; ^4^Foundation for Advanced Biomedical Research-Veneto Institute of Molecular Medicine (FABR-VIMM), Padova, Italy

## Abstract

**Objective:**

It has been shown that angiogenesis is a desirable treatment for patients with ischemic heart disease. We set out to investigate the impact of high-intensity interval training (HIIT) and berberine supplementation on the gene expression of angiogenesis-related factors and caspase-3 protein in rats suffering from myocardial ischemic-reperfusion injury.

**Methods:**

Fifty rats were divided into the following groups: (1) trained, (2) berberine supplemented, (3) combined, and (4) IR. Each cohort underwent five sessions of HIIT per week for a duration of 8 weeks followed by induction of ischemia. Seven days after completion of reperfusion, changes in the gene expression of angiogenesis-related factors and caspase-3 protein were evaluated in the heart tissue.

**Results:**

We observed a significant difference between four groups in the transcript levels of vascular endothelial cell growth factor (VEGF), fibroblast growth factor-2 (FGF2), and thrombospondin-1(TSP-1) (*p* ≤ 0.05). However, the difference in endostatin (ENDO) levels was not significant among the groups despite a discernible reduction (*p* ≥ 0.05). Moreover, caspase-3 protein and infarct size were significantly reduced in the intervention groups (*p* ≤ 0.05), and cardiac function increased in response to these interventions.

**Conclusion:**

The treatments exert their effect, likely, by reducing caspase-3 protein and increasing the expression of angiogenesis-promoting factors, concomitant with a reduction in inhibitors of the process.

## 1. Introduction

Previous works have shown that the reperfusion of blood in ischemic heart tissue is an effective method to treat acute myocardial infarction. However, reperfusion-induced oxidative stress can be detrimental and cause pathologic process of myocardial ischemia-reperfusion (IR) injury. Furthermore, ischemia-reperfusion injury is associated with microvascular dysfunction [1, 2], necessitating appropriate countermeasures to mitigate IR injury [3].

Angiogenesis, the formation of new blood vessels from preexisting ones, is indispensable for revascularization and cardiac remodeling following myocardial ischemia [4, 5]. Rehabilitation of the myocardium ischemic region involves the activation of several stimulatory and inhibitory modulators of angiogenesis; the most notable of which are vascular endothelial cell growth factor (VEGF), fibroblast growth factor-2 (FGF2), endostatin (ENDO), and thrombospondin-1 (TSP-1) [6]. VEGF is one of the major regulators of angiogenesis, which performs its duties by stimulating the proliferation and differentiation of vascular endothelial cells, increasing the permeability and reducing the apoptosis of endothelial cells through activating nitric oxide (NO). FGF2 stimulates angiogenesis through positive regulation of VEGF and dilation via NO [7]. Among the angiogenesis inhibitor proteins, thrombospondins play a key role in the heart remodeling and TSP-1 is one of the main physiological inhibitors of angiogenesis [8]. ENDO, a 20 kDa fragment of the C-terminal of type XVIII collagen, is also an inhibitor of angiogenesis, hampering endothelial cell proliferation, VEGF-induced migration, and endothelial duct formation [9].

Also, past studies have shown that prevention of apoptosis in endothelial cells improves angiogenesis in patients suffering from ischemia [10]. The most important caspase in the apoptotic endpoint is caspase-3 [11].

It has emerged that exercise is an effective approach to enhance angiogenesis and reduce caspase-3 [12]. Although regular endurance exercise interventions are invariably important for the preconditioning of the heart tissue, high intensity interval training is suggested to be a more effective approach which is recommended for patients with coronary heart disease [3].

On the other hand, berberine is an alkaloid in the form of yellow needle-shaped crystals, found in plants such as Berberis vulgaris [13]. To date, several studies have demonstrated that berberine exerts antiarrhythmic, antiapoptotic, and anti-inflammatory effects, culminating in a decrease in caspase-3 and the size of infarcted myocardium [13–15].

Wu et al. conducted a study to delineate the effect of exercise on the alterations of gene expression in angiogenesis context. Their findings demonstrated that three days of exercise in rats prior to induction of myocardial infarction leads to a significant increase of VEGF levels [16]. In this work, we investigated the effect of HIIT and berberine as preconditioning factors, solely or in combination, on changes in the expression of angiogenesis-promoting and angiogenesis-inhibiting molecular components (VEGF, FGF2, TSP-1, and ENDO) and caspase-3 protein one week after IR.

## 2. Methods

### 2.1. Subjects

Fifty 8-12-week-old male Wistar rats with a mean weight of 240 ± 20 g were acquired from the Pasteur Institute of Iran (PII). All investigations were carried out in accordance with the National Institutes of Health Guidelines for the Care and Use of Laboratory Animals, and the study protocol was approved by the animal's ethics committee at Bu-Ali Sina University of Hamedan (code: IR.BASU.REC.1398.041). The animals were divided into five groups (10 rats each) and kept in the animal house of Razi Research Center of Lorestan in a temperature of 22 ± 2°C, humidity of 60 ± 5%, and a 12-12 light-dark cycle with free access to food and water.

### 2.2. Preparation of Berberine Extract

Barberry roots were collected from wild specimens gathered from the city of South Khorasan (Iran). The roots (1 kg) were shade dried for two days at a temperature of 20 ± 5°C followed by being grinded with an electric grinder and completely powdered with a laboratory mill. Next, 100 g of the resulting powder was poured into a percolator. After 72 hours of shaking on a rotary shaker, the extract was passed through a filter paper and dissolved in a solvent (70% ethanol) utilizing a rotary machine which was used to condense the berberine extract. Finally, the obtained extract was stored in a refrigerator at 4°C. The concentration of berberine extract was determined by high-performance liquid chromatography (HPLC) (BFRL, China) and the standard commercial berberine (Berberine chloride, Sigma Aldrich). The column used in this machine was C18 with a diameter and height of 4.6 cm and 25 cm, respectively. The pump was SY-type which was also equipped with a UV/VIS detector. Moreover, measurements were performed at a wavelength of 330 nm with a flow rate of 1 mL/min, and the volume of each injection set was 25 *μ*L. The mobile phase was ethanol (50%) and water (50%) prepared for HPLC. The rats in supplemented and combined intervention groups received 10 mg/kg of berberine extract 5 days a week through gavage [14].

### 2.3. Exercise Protocol

The rats were transferred to Razi Research Center of Lorestan and were left undisturbed for a week to get habituated to their new conditions. Subsequently, the animals were made to run 10 minutes a day for a week at speeds of 10-20 m/min to be familiarized with the treadmill, prior to allocating them to the following groups at random: (1) trained group (IR after HIIT), (2) supplemented group (IR after berberine supplementation), (3) combined group (IR after HIIT and berberine supplementation), and (4) IR group (IR without intervention).

The exercise protocol included five sessions of interval running per week on a treadmill for 8 consecutive weeks. Each session consisted of 5 minutes of warming up, 5 minutes of cooling down period, and 30 minutes of interval running at speeds of 29-36 m/min (equivalent to 50-90% VO2max) on a fixed incline of one percent. In the first week, each session comprised 5 intervals, each of which consisted of 30-35 seconds of high-intensity running at a speed of 29 m/min with 1-minute rest time in between (active recovery at 20 m/min, equivalent to 50-60% VO2max). Each week, the number of intervals, their duration, and the running speed were gradually increased without changing the resting time. At the eighth week, the rats were subjected to twelve 75-second intervals at a speed of 36 m/min [17].

### 2.4. Ischemia-Reperfusion Injury

**Figure 1:**
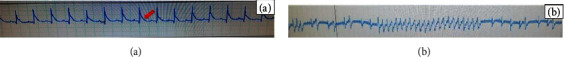
Electrocardiogram changes in ischemic rats. (a) ST segment elevation. (b) Premature ventricular contractions (PVC) and ventricular tachycardia (V Tach).

Forty-eight hours after the last exercise session and supplementation, the animals were anesthetized by intraperitoneal injection of ketamine (100 mg/kg) and xylazine (10 mg/kg), which took effect within 5 minutes. After that, fur was shaved, and the rats were fixed to a surgical bed for intubation, following which the animals were connected to a ventilator (Small Animal Ventilator, Harvard Model 683-USA) with a respiratory rate of 60-70 breaths per minute and a volume of 15 mL/kg. To stabilize body temperature, the surgical bed was equipped with a thermal pad with a mean temperature of 37 ± 1°C. Besides, occurrence of infarction was monitored by lead II recording, using a PowerLab electrocardiogram (ML750 Power Lab/4sp, AD Instruments). To induce the ischemia, a cross-cut of approximately 2 cm was made on the chest in the fourth left intercostal space to gain access to the heart. After gently rupturing the pericardium with small forceps, a silk thread (12 mm, USB6.0) was passed below the left anterior descending (LAD) coronary artery at a point of 2 mm under the left atrium and then pulled and tied to create experimental ischemia [3, 12]. The mean heart rate was calculated by an electrocardiogram (ECG). Also, ECG was used to monitor the successful blocking of LAD and changes in ECG, including ST-segment elevation (Figure 1(a)), premature ventricular contractions, and ventricular tachycardia in the electrocardiogram (Figure 1(b)). Thirty minutes after the blockage of LAD, the reperfusion maneuver was performed to restore blood flow to the myocardium. Then, the chest was closed by being sutured with silk thread (30 mm, USB 3.0), and tetracycline ointment was applied to the sutured area [3, 12].

### 2.5. Creatine Kinase-MB (CK-MB) Measurement

1 day after the surgery, anesthetization was performed with ketamine and xylazine (100 mg/kg and 10 mg/kg, respectively). In order for sampling the blood from the jugular vein, the rats were held by grasping the loose skin of the back firmly with fingers and elevating their heads. We shaved the hairs on the back of the neck and disinfected it with alcohol. By using a 1 mL syringe, a 25-gauge needle was inserted into the jugular vein through the pectoral muscle below the sternoclavicular junction. The blood was withdrawn slowly to avoid the collapse of these vessels, and approximately 1.5 mL blood was collected. After using a plastic tubing cutter to cut the syringe, the collected samples were transferred into heparin-coated capillary tubes and centrifuged at 4°C and 10,000 × g for 10 min. The plasma samples were stored at −80°C until the analyzing time. The blood plasma sample of each rat was prepared by a commercial kit (Pars Azmoon, Iran) according to the manufacturer's recommendations. In addition, the changes in the serum CK-MB levels were measured by a spectrophotometer (Cecil CE 7200).

### 2.6. Measurement of Echocardiography Indices

7 days after the surgery, anesthetization was performed with ketamine and xylazine (100 mg/kg and 10 mg/kg, respectively). Then, the chest was shaved, and the animal was placed on its back. Echocardiography was carried out by an echocardiography machine (M-Mode, Landwind Medical, p09 Specification, China) equipped with a 10 MHz transducer. Different echocardiographic indices were obtained according to the instructions of the American Society of Echocardiography [12]. Besides, the stroke volume (SV) was calculated from the difference between the end-systolic volume and end-diastolic volume (SV = diastolic volume − systolic volume), and the cardiac output is a product of the heart rate and the stroke volume (CO = stroke volume × heart rate). Also, the percent of muscle shortening (% FS) and injection fraction (% EF) was calculated as follows: %FS = ((LVEDD − LVESD)/LVEDD) × 100; %EF = (stroke volume/diastolic volume) × 100 [18].

### 2.7. Collecting Heart Tissues

After echocardiography, the chest was opened, and the heart was quickly excised. Next, the heart was rinsed with distilled water, snap frozen upon removal of atria and vascular root, and weighed. In addition, the heart weight (HW) to body weight (BW) ratio and HW to tibia length (TL) ratio were calculated [19]. The heart biopsy was performed at 1 mm far from the damaged area of the rat's heart [12]. After that, the tissue samples were quickly frozen in liquid N2 for further analyses. The duration of the process was less than 2 min. Finally, the target tissues were stored at -80° C.

### 2.8. Caspase-3 Protein

Total lysates were prepared from left ventricular tissue of the infarct area one week after reperfusion. Capsase-3 levels were measured by ELISA kit (ZellBio, Germany, Cat#GB-326-1) as per the manufacturer's recommendations. In brief, 40 *μ*L of each lysate, along with standard caspase-3, was incubated with caspase antibody for 60 minutes. Following this incubation, the wells were washed and incubated with streptavidin-HRP for 60 minutes at 37°C. After that, the plates were washed again, and chromogenic substrate was added to each well which incubated for 10 minutes followed by addition of stop solution. The optical density of each well was read on an ELISA plate reader.

### 2.9. Real-Time Polymerase Chain Reactions

RNA was extracted using tissue/Max Spin super RNA extraction kit (Maxcell, Iran) based on the manufacturer's recommendations. cDNA was also synthesized using a cDNA Synthesis Kit (Yekta Tajhiz, Iran). Also, PCR primers were purchased from SinaClon (Iran) (Table 1). PCR reactions were set up as follows: 1 *μ*L of SYBR Green master mix (Yekta Tajhiz, Iran) was mixed with 5 *μ*L of RNase-free water. 1 *μ*M of both forward and reverse primers and 100 ng of cDNA templet were added to the above mix and subjected to amplification cycles. The *β*-actin mRNA was used as the reference gene. The target gene expression was quantified using the formula 2^−ΔΔCT^ [12].

### 2.10. Infarct Size Measurement

2 mm slices were made from the left ventricle (from base to apex) of the frozen hearts by graded molds. Next, the container containing the tetrazolium solution was inserted into the bain-marie at 37° C. Then, the heart was immersed in a bain-marie for 15–20 minutes. It is observed that the infarct areas due to necrosis and the loss of intracellular dehydrogenase enzymes could not react with tetrazolium, and, as a result, these areas turn yellow to white. To increase the contrast of dark red and white areas, the slices were fixed in 10% formalin for 48 h. After this period, scans were obtained from the tissues. Then, with Photoshop software (Adobe Photoshop, 2019), the surface area of necrotic or infarct zones were calculated as a percentage of the infarcted left ventricle [12].

### 2.11. Statistical Analysis

Statistical analyses and comparisons were performed in SPSS version 23. The comparison between the groups was performed in terms of Mean ± SEM of the results at *p* ≤ 0.05 significance level. After establishing the normality of data distribution by Shapiro-Wilk test, they were further analyzed with one-way ANOVA and Turkey's post hoc test [3].

## 3. Results

### 3.1. General Characteristics

Measurement of the heart weights did not reveal any difference amongst the cohorts (Table 2). At the completion of the treatment, we detected reduced body mass in all experimental groups compared to IR group (*p* = 0.01) (Table 2). The heart weight to body weight ratio, however, was only increased in the trained and the combined groups in comparison with IR rats (*p* = 0.01) (Table 2).

### 3.2. Echocardiographic Data

Stroke volume (SV), cardiac output (CO), fractional shortening (FS), and ejection fraction (EF) had a significant increase in three intervention groups compared to IR group (*p* = 0.01). EF was 51.74 ± 0.06% in IR group and 63.5 ± 0.9, 63.4 ± 0.4, and 64.7 ± 0.9 in three intervention groups (Table 3).

### 3.3. Creatine Kinase-MB

**Figure 2:**
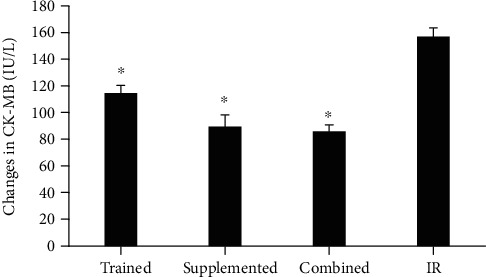
Creatine kinase-MB changes in different groups after 1 day of ischemia-reperfusion. ^∗^Significant difference in comparison with IR group. *p* < 0.05.

As depicted in Figure 2, serum level of CK-MB was increased in IR group, and CK-MB elevations were more modest in intervention groups than those in IR group at day one of postreperfusion (*p* = 0.01) (Figure 2).

### 3.4. Caspase-3

**Figure 3:**
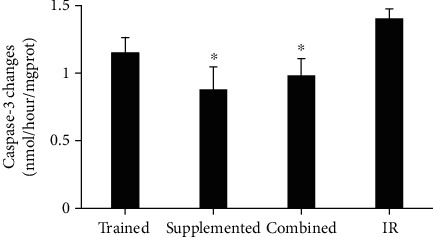
Caspase-3 changes in different groups after 7 days of ischemia-re in rat heart tissue. ^∗^Significant of difference in comparison with IR group. *p* < 0.05.

ELISA analysis revealed a significant reduction in Caspase-3 levels in supplemented and combined groups compared to IR group (*p* = 0.01) (Figure 3).

### 3.5. Gene Expressions in the Border Zone Left Ventricular

**Figure 4:**
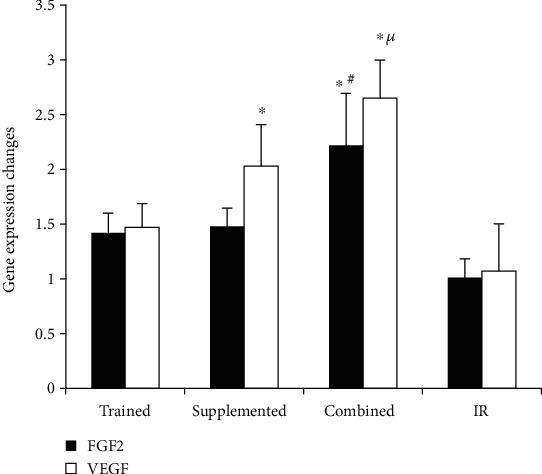
Fibroblast growth factor and vascular endothelial growth factor gene expression changes in the heart tissue. ^∗^Significant difference in comparison with IR group. ^**#**^Significant difference in comparison with trained and supplemented group. ^*μ*^Significant difference in comparison with trained group. *p* < 0.05.

Q-PCR analysis revealed some alterations in the expression of angiogenesis-related genes in myocardial tissues among different experimental rat groups. In this analysis, VEGF transcript levels were elevated in supplemented group compared to that of IR group. Also, an increase in VEGF was observed in the combined group compared to the trained and IR groups (*p* = 0.001). Interestingly, elevated levels of FGF2 transcripts were only detected in the myocardial tissues of the rats subjected to this later regiment (*p* = 0.001) (Figure 4).

**Figure 5:**
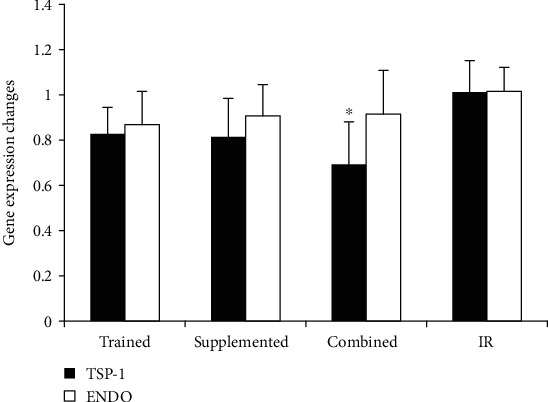
Endostatin and thrombosponidin-1 gene expression changes in the heart tissue. ^∗^Significant difference in comparison with IR group. *p* < 0.05.

Analysis of angiogenic inhibiting factors showed that neither TSP-1 nor ENDO was reduced at mRNA level by either training or supplement treatment separately. The only significant reduction in TSP-1 transcript was discerned in the rat under combined treatment (*p* = 0.01) (Figure 5).

In conclusion, these data suggest that although training or berberine treatment separately can support angiogenesis in ischemic myocardial tissue, the combination of them synergizes to exert a larger effect.

### 3.6. Infarct Size

**Figure 6:**
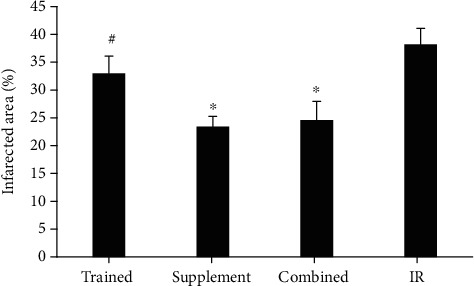
Infarct size area in different groups after 7 days of ischemia-reperfusion in the heart tissue of the rats. ^∗^Significant difference in comparison with IR group. ^#^Significant difference in comparison with supplement and combined groups. *p* < 0.05.

The size of the infarct area was smaller in supplemented and combined cohorts of rats (supplemented 23.4 ± 1.85% and combined 24.6 ± 3.36%) with respect to that of IR (38.2 ± 2.86%) and trained rats (33 ± 3.08%) (Figure 6).

## 4. Discussion

Our results indicated a higher heart to body weight ratio in the trained and combined treated rats as opposed to the IR group. Also, despite the decrease in heart rate in the intervention groups compared to the IR, but with an increase in stroke volume, an improvement in heart function was shown.

The infarct size is an appropriate indicator of cardiac resistance to IR injury [3], and the role of apoptosis in regulating infarct size has been shown before [20]. In this regard, there is an elevation of caspse-3 expression a few hours after reperfusion, which lasts for several weeks [20, 21].

This study found that the combination of HIIT and berberine, as preconditioning factors, reduced caspase-3 and infarct size in intervention groups. In support of our findings, there is evidence that angiogenic factors, such as VEGF and FGF2, impact apoptosis by regulating the expression of antiapoptotic proteins such as BCL-2, surviving, and XIAP, through activation of phosphatidylinositol 3-kinase-mediated Akt )PI3K/Akt( pathway and increasing nitric oxide [10, 22] . Also, angiostatic factors such as THPS-1 and ENDO are known to activate caspase-3 and apoptosis through dephosphorylation of endothelial nitric oxide synthase, depletion of BCL- and BCL-XL and activation of p38 group of mitogen-activated protein (MAP) kinases (P38 MAPK pathway) [10, 21].

Creatine kinase-MB is a biomarker of heart damage due to its highly regulated balance in the cardiac tissue [23]. The serum level of CK-MB reaches its peak 24 hours after ischemia and returns to basal level within 72 hours [24]. High serum CK-MB levels in our model is indicative of damage to the cardiac tissue. The protective role of both HIIT and berberine treatment, seperataley or in combination, against the cardiac damage is shown by reduced serum levels of creatine kinase-MB in our study.

Our findings also uncovered regulations exerted by different treatments on the gene expression of angiogenesis factors in the injured tissue. The increase of VEGF mRNA to a greater extent in the supplemented and combined groups indicates that exercise can be more effective if combined with berberine treatment, in line with Yardley et al. [25] observations. Our data, however, are in contrast to that of Nazarali et al. [26]. This inconsistency is likely to stem from the presence or lack of ischemia-reperfusion injury in the rats, the type and intensity of exercises, the age of the animals, and the target tissue. Hemodynamic angiogenesis stimuli, such as physical activity, are known to increase the level of nitric oxide by creating shear stress and activating ion channels (e.g., potassium channels). Nitric oxide, in turn, elevates the myocardial VEGF levels, thereby promoting angiogenesis [26, 27].

Although the role of fibroblast growth factor family in angiogenesis of the heart has not fully understood yet, FGF2 is a known stimulator of endothelial cell proliferation and the physical organization of endothelial cells [28, 29]. In our experimental IR condition, the detection of elevated FGF2 in the surrounding tissue upon ischemia is indicative of its role in promoting angiogenesis in ischemic tissue. Considering that hypoxia, NO, and NADPH factors also regulate FGF2 expression in the repairing tissues [28, 30, 31], the significant increase in the myocardial FGF2 in the combined group suggests that the surrounding tissue of damaged myocardium has probably healed sooner and has experienced improved blood reperfusion. Some studies have reported that an increased FGF2 expression occurs at high volumes of exercise in subjects with high body fat [32]. Furthermore, although berberine supplementation increases angiogenesis [15], the dose of berberine in our study may not have been sufficient to increase FGF2 significantly.

In addition to regulating the rate of blood flow during intense exercise, endostatin can act as both pro- and antiangiogenic factor through stimulation of VEGF-dependent endothelial cells proliferation and induction of apoptosis, respectively [27, 33]. In line with Brixius et al. report [33], we did not find any changes in myocardial expression of endostatin in ischemic tissues.

Our results demonstrated a significant reduction in levels of TSP-1 in the ischemic tissue of the rats treated with combination of berberine and HIIT. TSP-1 exerts its antiangiogenic effect through several mechanisms including, but not limited to, inhibition of endothelial cell cycle progression and mobilization, blocking VEGF and extracellular signal-regulated kinase (ERK) signaling pathways, and altering the function of NO and apoptosis in endothelial cells [8, 34, 35]. Given the natural increase of TSP-1 level after ischemia in the area near to the site of infarction [8], which leads to a limited spread of granulation tissue, our observations indicate the effectiveness of this combined treatment in improving wound healing process. Berberine has also been shown to promote angiogenesis in the ischemic myocardial tissue through activation of phosphatidylinositol 3-kinase-mediated Akt- (PI3K/Akt-) dependent signaling and AMPK in endothelial cells [15].

## 5. Conclusion

In brief, our study demonstrates that the combined use of berberine extract and HIIT can lead to significant changes in the myocardial levels of angiogenic (VEGF and FGF2), angiostatic (TSP-1) factors and caspase-3 protein, 7 days after reperfusion. However, it can be said that HIIT and berberine, solely or in combination, reduce caspase-3 and the size of myocardial infarction. Nevertheless, when these interventions combined, they have a greater effect on the expression of the angiogenesis gene in the infarcted heart. These changes were manifested as an increase in the levels of the former and a decrease in the levels of the later agents. We suggest that this combined intervention can be considered as an important precautionary method in myocardial infarction events.

## Figures and Tables

**Table 1:** Primer sequence of selected angiogenic and angiostatic genes.

Gene	Forward	Reverse
VEGF	TGTGTGTGTGTATGAAATCTGTG	GCAGAGCTGAGTGTTAGCAA
FGF	ACGGCTGCTGGCTTCTAAGTG	AGTTCGTTTCAGTGCCACATACC
TSP-1	TCGCAAAGTGACGGAAGAGAAC	ATTGGAGCAGGGCATGATGG
ENDO	ACTGCCTGGATGAAGAAGATGATG	CGTAGATATGTCTCCTGCCTGTG
*β*-Actin	GTAACCCGTTGAACCCCATT	CCATCCAATCGGTAGTAGCG

*β*-Actin: beta-actin; ENDO: endostatin; FGF: fibroblast growth factor; TSP-1: thrombospondin-1; VEGF: vascular endothelial growth factor.

**Table 2:** Demographic characteristics of the rats in different groups.

Groups	Trained	Supplemented	Combined	IR
Number	10	10	10	10
Age (week)	8-10	8-10	8-10	8-10
Heart rate (bpm)	376 ± 3.02	378 ± 1.93	375 ± 2.32	368 ± 2.48
Heart weight (g)	0.91 ± 0.04	0.90 ± 0.06	0.90 ± 0.08	0.78 ± 0/01
Body weight (g)	266 ± 20^∗^	277 ± 50^∗^	265 ± 10^∗^	319 ± 70
Heart weight/body weight (g)	0.003 ± 0.0001^∗^	0.002 ± 0.0001	0.002 ± 0.0003^∗^	0.002 ± 0.0004
Heart weight/tibia length (g/mm)	0.023 ± 0.003	0.023 ± 0.002	0.022 ± 002	0.019 ± 001

Data are presented as Mean ± SEM. ^∗^Significant difference in comparison with IR group. *p* < 0.05.

**Table 3:** The mean cardiac echocardiography indexes of the rats in different groups at 1-week post IR.

Groups	Train	Supplemented	Combined	IR
SV (mL)	187.77 ± 6.92^∗^	184.10 ± 15.00^∗^	195.21 ± 16.43^∗^	128.45 ± 6.42
CO (mL/min)	70 ± 27^∗^	69 ± 13^∗^	73 ± 16^∗^	57 ± 12
EF (%)	63.5 ± 0.9^∗^	63.4 ± 0.4^∗^	64.7 ± 0.9^∗^	51.74 ± 0.6
FS (%)	35 ± 0.6^∗^	35 ± 0.7^∗^	36 ± 0.2^∗^	27 ± 0.8

SV: stroke volume; CO: cardiac output; EF: left ventricular ejection fraction; FS: fractional shortening. Values are means ± standard deviations. The data are mean ± sem. ^∗^Sign of significant difference with IR group. ^∗^*p* ≤ 0.05.

## Data Availability

The datasets generated/analyzed during the current study are available.

## Abbreviations

IR: Ischemia-reperfusion
HIIT: High-intensity interval training
VEGF: Vascular endothelial cell growth factor
FGF2: Fibroblast growth factor
TSP-1: Thrombospondin-1
ENDO: Endostatin
PII: Pasteur institute of Iran
HPLC: High-performance liquid chromatography
PI3K/Akt: Phosphatidylinositol 3-kinase-mediated Akt
P38 MAPK pathway: p38 group of mitogen-activated protein (MAP) kinases
LAD: Left anterior descending
ERK: Extracellular signal-regulated kinase
*β*-Actin: Beta-actin
PVC: Premature ventricular contractions
V Tach: Ventricular tachycardia.
